# Distribution of Modic changes in patients with low back pain and its related factors

**DOI:** 10.1186/s40001-019-0393-6

**Published:** 2019-10-09

**Authors:** Yufeng Chen, Jie Bao, Qi Yan, Cenhao Wu, Huilin Yang, Jun Zou

**Affiliations:** 1 Department of Orthopaedic Surgery, Jiangsu Taizhou People’s Hospital, Taizhou, 225300 Jiangsu China; 2 Department of Orthopaedic Surgery, The First Affiliated Hospital of Soochow University, 188 Shizi St., Suzhou, 215006 Jiangsu China; 3 School of Physical Education and Sports Science, Soochow Univeristy, Suzhou, 215021 Jiangsu China

**Keywords:** Modic changes, Types, Related factors, LBP

## Abstract

**Background:**

To summarize the clinical distribution of Modic changes in patients with low back pain and explore the related factors.

**Methods:**

A total of 153 patients were enrolled. Gender, age, disk degeneration, herniation, involved segments, lumbar lordosis angle, and endplate concave angle were recorded, respectively. Patients were divided into two or more groups according to a different classification. The relevant factors were studied with a multivariate logistic regression analysis to analyze their correlation.

**Results:**

A total of 35 patients with type I changes, 110 patients with type II changes, and 8 patients with type III changes. In total, 204 disks were found with Modic changes, L1/2 (10 disks), L2/3 (18 disks), L3/4 (17 disks), L4/5 (76 disks), and L5/S1 (81 disks). Type I changes were distributed mainly under the age of 50. Multivariate regression showed that gender, age, disk degeneration, lumbar lordosis, L4/5 segment lordosis angle, and L5 lower endplate concave angle were related with different types of Modic changes. The regression equation *Y* = 2.410 − 1.361*S *− 0.633*A *− 0.654*P* + 1.106*L *− 0.990*D* (*Y* means type I changes, *S* means gender, *A* means age, *P* means disk degeneration, *L* means L4/5 segment lordosis angle, and *D* means L5 upper endplate concave angle). The OR values were *S* = 0.256, *A* = 0.531, *P* = 0.520, *L* = 3.022, *D* = 0.372, respectively.

**Conclusions:**

Type II changes are the most common, followed by type I. Modic changes mostly occur in L4/5 and L5/S1; young, male, lower-grade disk degeneration, normal physiological curvature of the lumbar spine, and normal endplate concave angle were associated with type I changes; gender and lumbar curvature were the most relevant factors for different types.

## Background

Low back pain (LBP) is a common disease, and up to 80% of adults have suffered from it during their lifetime [1]. The lumbar degenerative disease is one of the most important causes for LBP. Endplate locates between the disk and vertebral and intimately connects to the disk. As endplate is very thin, it is difficult to measure through regular imaging methods. With the continuous development of magnetic resonance imaging (MRI) technology, the relationship between endplate, vertebral, and LBP has received a lot of attention. Modic changes are a common abnormal signal change in lumbar MRI, reflecting the microscopic changes in tissue biochemistry in the endplate, which is an early manifestation of endplate degeneration. The concept of Modic changes has been in existence for more than 30 years; however, the specific mechanism still remains unknown. The relationship between it and LBP has been widely studied, and most scholars believe that there is a connection [2–6]. Toyone et al. [7] studied 74 LBP patients, 73% (27/37) of patients with type I changes, while only 11% (4/37) with type II changes. They concluded that type I changes are more closely related to LBP. Ohtori et al. [8] found that the number of inflammatory factors such as tumor necrosis factor (TNF) and protein gene product 9.5 (PGP 9.5) was significantly increased in patients with type I changes. Different types of Modic changes have different pathological changes and therefore have different effects on LBP and surgical success [9–11]. However, no clinical study has been conducted to investigate the relevant factors associated with different types of Modic changes. This study retrospectively analyzed 153 patients with Modic changes and LBP in order to summarize the clinical distribution of Modic changes on patients with low back pain and explore the related factors.

## Materials and methods

### General information

Patients who took lumbar MRI for LBP in our hospital from January 2015 to December 2017 and diagnosed with endplate Modic changes were enrolled. According to the following inclusion/exclusion criteria, 153 patients were included in the study.

#### Inclusion criteria

Patient inclusion criteria are as follows: (1) a typical endplate Modic changes on T1-weighted image and T2-weighted image and (2) clear and complete imaging data.

#### Exclusion criteria

Exclusion criteria are: (1) fresh compression fracture of the lumbar vertebral; (2) lumbar spondylolisthesis, scoliosis; (3) tuberculosis, tumor, etc.; (4) Schmorl’s node; and (5) a history of back surgery.

### Modic changes

**Fig. 1:**
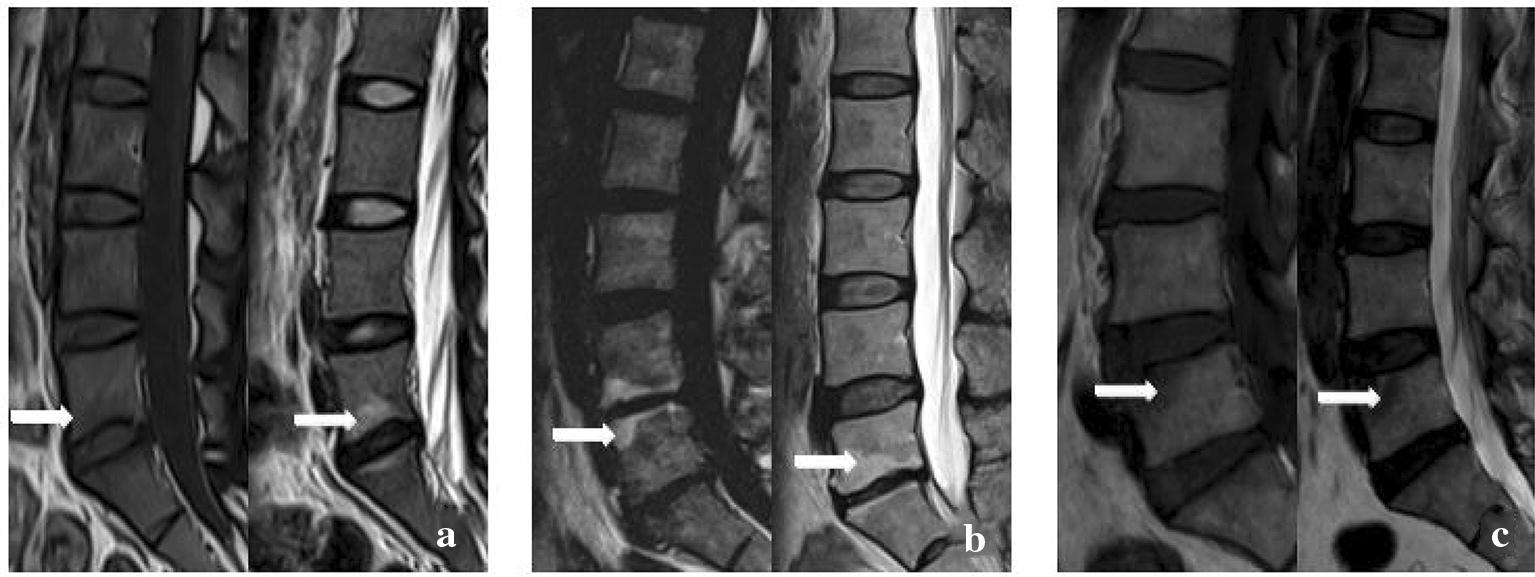
Typical Modic changes. **a** Type I changes; **b** type II changes; **c** type III changes

According to the classification criteria proposed by Modic et al. [12], Modic changes were classified into three types: type I—hypointense on T1WI and hyperintense on T2WI; type II—hyperintense on T1WI and isointense or mildly hyperintense on T2WI; and type III—hypointense on both T1WI and T2WI (Fig. 1).

### Age

According to the classification raised by WHO in 2012, the patients were divided into three groups: young group—under 44 years old; middle-aged group—between 45 and 59 years; elderly group—over 60 years.

### Intervertebral disk degeneration

**Fig. 2:**
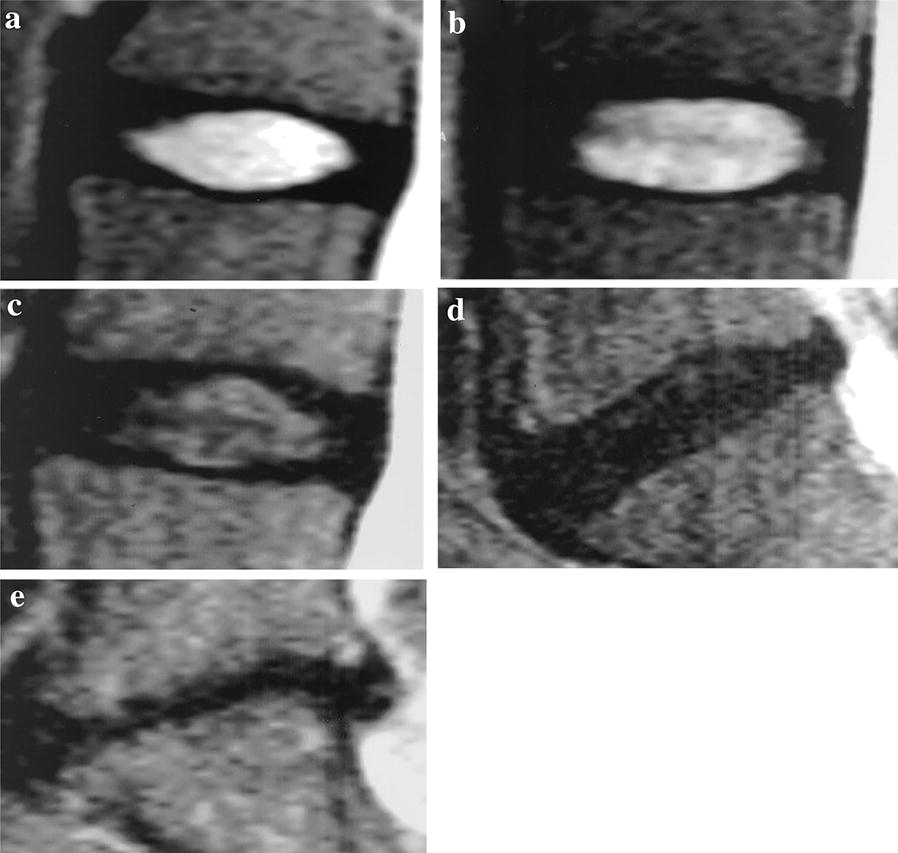
Typical Pfirrmann classification. **a** Grade I; **b** grade II; **c** grade III; **d** grade IV; **e** grade V

According to the classification raised by Pfirrmann et al. [13], all cases of intervertebral disk degeneration were divided into five grades (Table 1, Fig. 2).

**Table 1:** Classification of intervertebral disk degeneration on MRI (Pfirrman scale)

Grades	Structures	Distinction between nucleus and annulus	Signal intensity	Disk height
I	Bright white, homogeneous	Clear	Hyperintense, isointense to cerebrospinal fluid	Normal
II	Inhomogeneous with or without horizontal bands	Clear	Hyperintense, isointense to cerebrospinal fluid	Normal
III	Gray, inhomogeneous	Unclear	Intermediate	Normal to slightly decreased
IV	Gray to black, inhomogeneous	Lost	Intermediate to hypointense	Normal to moderately decreased
V	Black, inhomogeneous	Lost	Hypointense	Collapsed

### Disk herniation

**Fig. 3:**
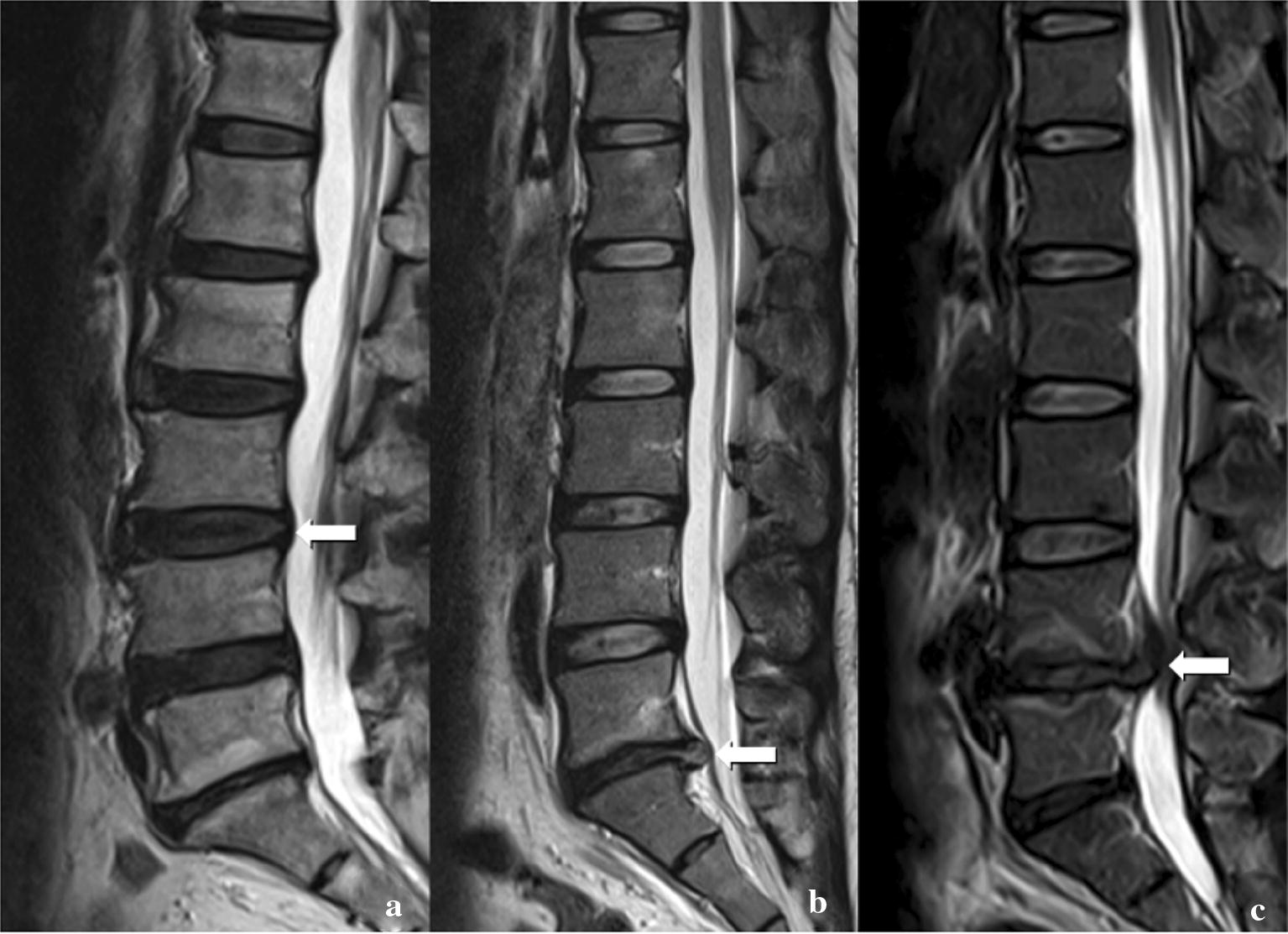
Typical classification of intervertebral disk degeneration. **a** Protruded; **b** extruded; **c** sequestrated

According to the classification raised by the American Association of Orthopedic Surgeons, all cases of disk herniation were divided into three types: protruded, extruded, and sequestrated (Fig. 3).

### Lumbar curvature

**Fig. 4:**
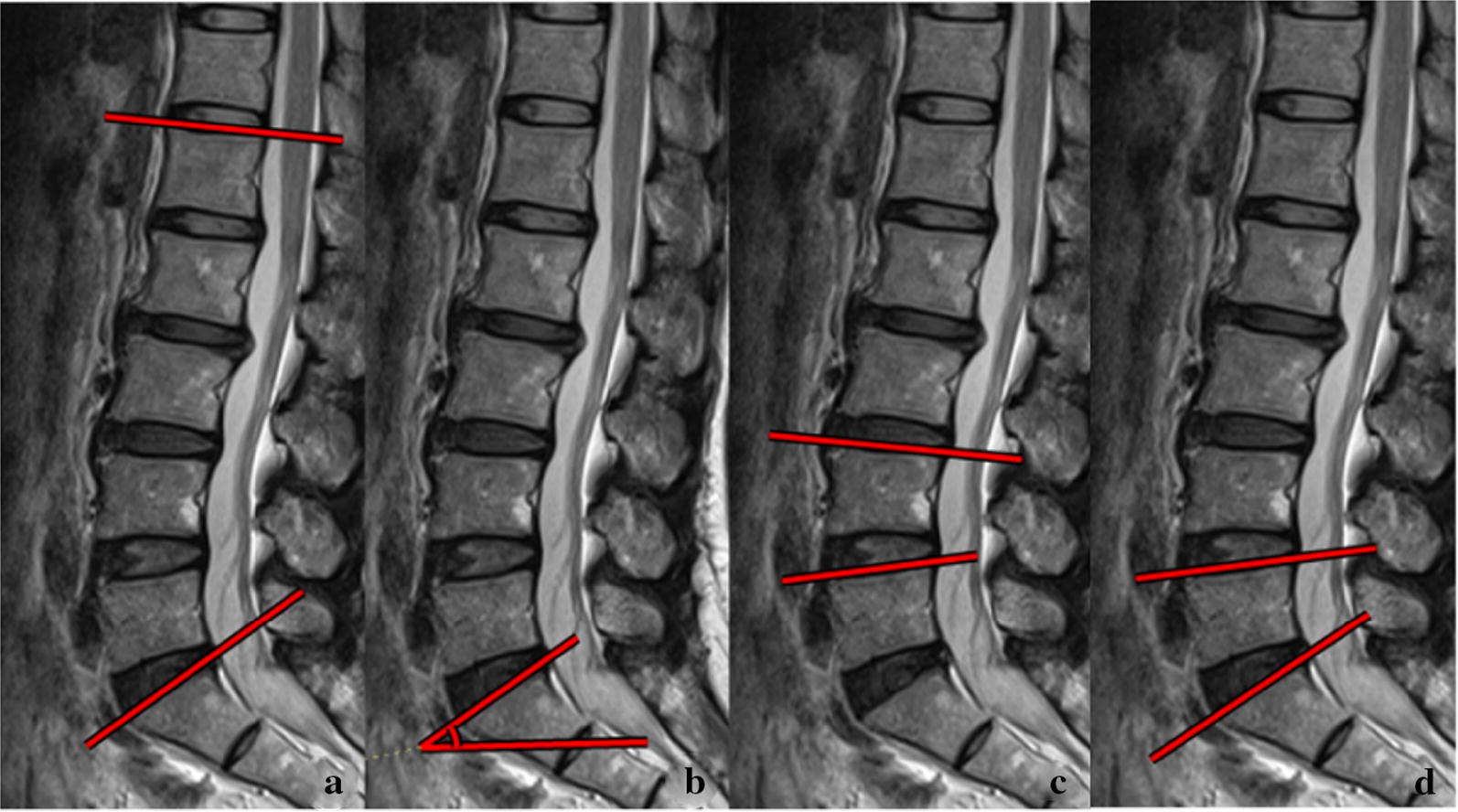
The method of measuring lumbar lordosis. **a** LL; **b** SS; **c** L4/5 lordosis; **d** L5/S1 lordosis

Lumbar lordosis (LL), sacral slope (SS), the lordosis angle of the L4/5 vertebral segment, and the lordosis angle of the L5/S1 vertebral segment were measured (Fig. 4).

### Concave angle of vertebral endplate

**Fig. 5:**
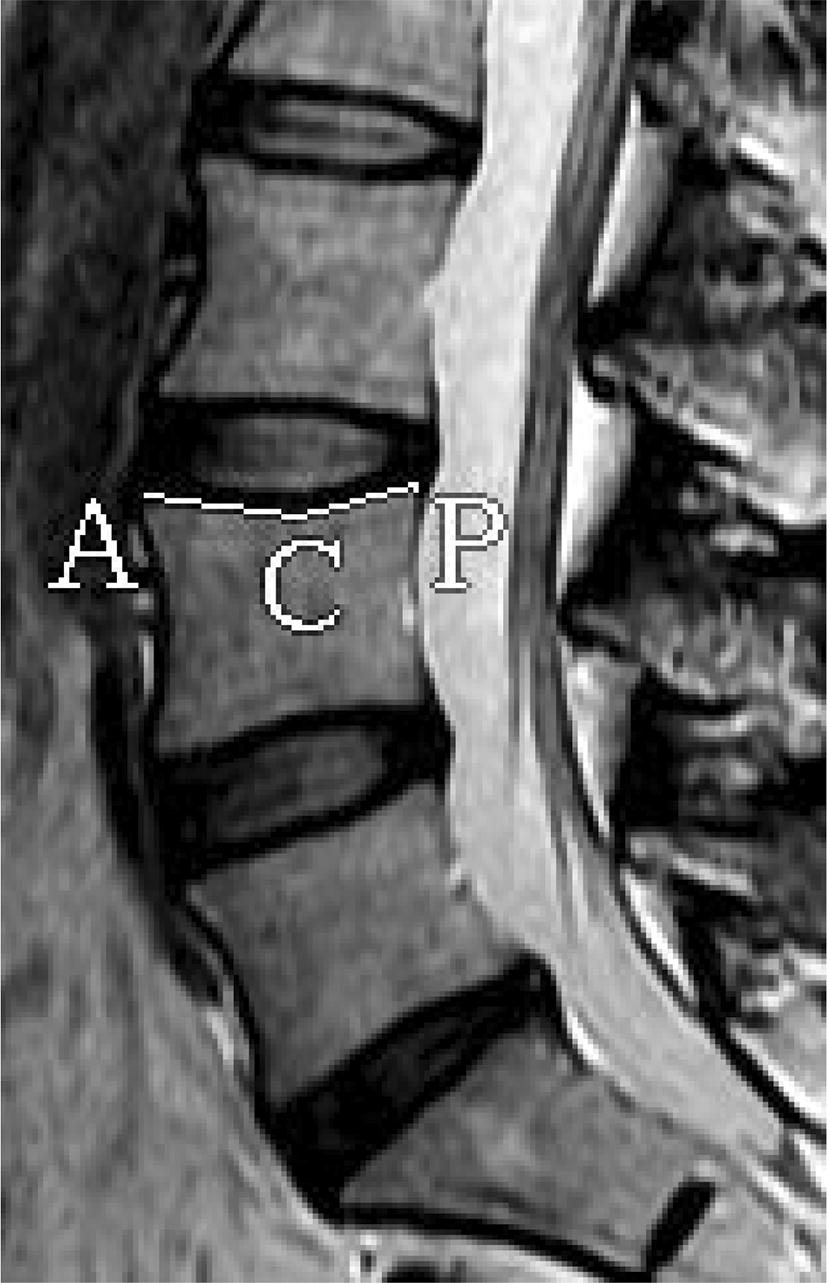
The method of measuring CAVE. ∠ACP: L4 upper CAVE

The concave angles (CAVE) of the L4 lower endplate, the L5 upper endplate, and the L5 lower endplate were measured (Fig. 5).

## Data analysis

All clinical and imaging data were evaluated by an orthopedic surgeon and a radiologist. Data analysis was performed by SPSS 20.0 statistical software. Firstly, the univariate Chi-square test was used to study the individual factors, and *P* < 0.05 indicated statistical significance. Factors with statistical significance were included in a binary logistic regression analysis to study their associations with the different types of Modic changes.

## Results

### Distribution of different types

There were a total of 35 patients with type I changes, 110 patients with type II changes, and 8 patients with type III changes. The incidence rate was 22.9%, 71.9%, and 5.2%, respectively. In total, 204 disks were found with Modic changes, and the involved segments were: L1/2 (10 disks), L2/3 (18 disks), L3/4 (17 disks), L4/5 (76 disks), and L5/S1 (81 disks). Patients under 50 years were more likely to have type I changes, and those over 50 years were more likely to have type II changes.

### Relationship between types and age

There were 15 patients in the young group, 14 patients in the middle-aged group, and 6 patients in the elderly group with type I changed; there were 23 patients in the young group, 50 patients in the middle-aged group, and 37 patients in the elderly group with type II changes. There was a statistical difference among the three groups (Table 2).

**Table 2:** Distribution of different types of Modic changes with age

Groups	Modic changes		Total	*χ* ^2^	*P*
	Type I	Type II			
Youth group	15	23	38	7.495	0.024*
Middle-age group	14	50	64		
Elderly group	6	37	43		
Total	35	110	145		

*statistically significant difference (*p* < 0.05)

### Relationship between types and gender

Patients with type I changes consisted of 23 men and 12 women; patients with type II changes consisted of 49 men and 61 women. There was a statistical difference between two groups (Table 3).

**Table 3:** Distribution of different types of Modic changes with gender

Groups	Modic changes		Total	*χ* ^2^	*P*
	Type I	Type II			
Male	23	49	72	4.760	0.029*
Female	12	61	73		
Total	35	110	145		

*statistically significant difference (*p* < 0.05)

### Relationship between types and affected segments

When two or more segmental were involved, the segment with the largest change area ratio (abnormal signal area/total vertebral body area) was considered as the affected segment. There were 27 L1/4 segments, 55 L4/5 segments, and 71 L5/S1 segments. For patients with type I changes, there were 6 L1/4 segments, 14 L4/5 segments, and 15 L5/S1 segments; for patients with type II changes, there were 13 L1/4 segments, 41 L4/5 segments, and 56 L5/S1 segments. There was no significant difference between the three groups (Table 4).

**Table 4:** Distribution of different types of Modic changes with involved segments

Groups	Modic changes		Total	*χ* ^2^	*P*
	Type I	Type II			
L1/4	6	13	27	0.978	0.613
L4/5	14	41	55		
L5/S1	15	56	71		
total	35	110	145		

### Relationship between types and intervertebral disk degeneration

When two or more segmental were involved, the segment with the largest change area ratio (abnormal signal area/total vertebral body area) was considered as the affected segment. For patients with type I changes, there were 7 grade II, 10 grade III, 12 grade IV, and 6 grade V; for patients with type II changes, there were 6 grade II, 19 grade III, 31 grade IV, and 54 grade V. There was a statistical difference among all groups (Table 5).

**Table 5:** Distribution of different types of Modic changes with Pfirrmann classification

Groups	Modic changes		Total	*χ* ^2^	*P*
	Type I	Type II			
II	7	6	13	14.843	0.002*
III	10	19	29		
IV	12	31	34		
V	6	54	60		

*statistically significant difference (*p* < 0.05)

### Relationship between types and disk herniation

For patients with type I changes, 23 bulged, 11 protruded, and 1 extruded; for patients with type II change, 58 bulged, 46 protruded, and 6 extruded. Patients with type I changes had higher proportions of bulged hernia but lower proportions of protruded and extruded hernia, but there was no statistical difference among the groups (Table 6).

**Table 6:** Distribution of different types of Modic changes with disk degeneration

Groups	Modic changes		Total	*χ* ^2^	*P*
	Type I	Type II			
Protruded	23	58	81	1.902	0.386
Extruded	11	46	57		
Sequestrated	1	6	7		
Total	35	110	145		

### Relationship between types and lumbar curvature

Lumbar lordosis (LL), sacral slope (SS), and the lordosis angles of the L4/5 and L5/S1 vertebral segments were divided into two groups according to the mean degree. The mean degree of LL was 34.7°, SS was 29.7°, and L4/5 and L5/S1 vertebral segment lordosis angles were 9.4° and 17.2°, respectively. Patients with type I changes had larger LL and L4/5 vertebral segment lordosis angle, with statistical differences. There was no significant difference for SS and L5/S1 vertebral segment lordosis angle (Table 7).

**Table 7:** Distribution of different types of Modic changes with lumbar lordosis angle

Groups	Modic changes		Total	*χ* ^2^	*P*
	Type I	Type II			
LL < 35°	12	61	73	4.760	0.029*
≥ 35°	23	49	72		
SS < 30°	13	59	72	2.889	0.089
≥ 30°	22	51	73		
L4/5 lordosis < 10°	12	62	74	5.179	0.023*
≥ 10°	23	48	71		
L5/S1 lordosis < 18°	20	55	75	0.543	0.401
≥ 18°	15	55	70		

*statistically significant difference (*p* < 0.05)

### Correlation between types and CAVE

CAVE was divided into two groups according to the mean degree. The mean degree of L4 lower was 164.8°, that of L5 upper was 168.6°, and that of L5 lower was 165.2°. Patients with type I changes had a smaller degree than type II changes for each CAVE. However, there was a statistical difference only for L5 lower CAVE (Table 8).

**Table 8:** Distribution of different types of Modic changes with endplate concave angle

Groups	Modic changes		Total	*χ* ^2^	*P*
	Type I	Type II			
L4 lower < 165°	19	47	66	1.430	0.232
≥ 165°	16	63	79		
L5 upper < 169°	20	52	72	1.035	0.309
≥ 169°	15	58	73		
L5 lower < 166°	21	52	73	5.179	0.023*
≥ 166°	14	58	72		

*statistically significant difference (*p* < 0.05)

### Univariate analysis

Univariate Chi-square test showed that age, gender, disk degeneration, lumbar lordosis, L4/5 segment lumbar lordosis, and L5 lower CAVE were associated with different types. After correlation analysis of the above factors by binary logistic regression analysis, no significant correlation was found between lumbar lordosis and types. Binary logistic regression analysis shows that the regression equation is *Y* = 2.410 −1.361*S* −0.633*A* −0.654*P* + 1.106*L* −0.990*D* (*Y* means type I changes, *S* means gender, *A* means age, *P* means the disk degeneration, *L* means L4/5 segment lumbar lordosis, and *D* means L5 upper endplate concave angle. The OR values are *S* = 0.256, *A* = 0.531, *P* = 0.520, *L* = 3.022, *D* = 0.372, respectively (Table 9).

**Table 9:** Multivariate logistic regression correlation analysis

	*P*	EXP	95% CI
Age	0.035	0.531	0.295–0.956
Gender	0.005	0.256	0.100–0.159
Pfirrmann	0.004	0.520	0.332–0.814
L4/5 lordosis	0.020	3.022	1.187–7.690
L5 lower CAVE	0.035	0.372	0.148–0.934

## Discussion

### Distribution of Modic changes among patients with LBP

Our results showed that type II changes are most common and the proportion is 71.9% for type II, 22.9% for type I, and 5.2% for type III. Modic changes mainly occur in L4/5 and L5/S1 segments, accounting for 77% of all cases. These findings are consistent with previous reports [12, 14]. Many studies had demonstrated that Modic changes mostly occurred in L4/5 and L5/S1 segments, where lumbar suffered maximum stress [9, 15]. Patients under 50 are more likely to have type I changes, and those over 50 are more likely to have type II changes. The incidence of Modic changes in general population is about 6% [6, 16], and the incidence in LBP population is 18% to 62% [7, 9].

### Age and types

Our results show that age is one of the factors associated with different types. Modic changes are degenerative disease, with a higher incidence rate over 45 [6]. In this study, young patients are closely associated with type I changes, and thus, we hypothesize that there may a transformation between two types, type I can convert to type II with the increasing of age. Braithwaite et al. [17] believed that different types may be different pathological processes of the same disease, and all types can be inter-transformable. Modic et al. [12] conducted a 3-year follow-up study and found that 5 of 6 type I changes converted to type II, while 10 type II changes remained unchanged. They, therefore, concluded that type I changes are an unstable state and type II changes are relatively stable. A similar study conducted by Mitra et al. [18] found that 48 patients with type I changes, 18 completely transformed into type II, 7 partially transformed into type II, 19 remained unchanged with expanded abnormal signal range, and 4 remained unchanged. They considered that type I changes are a dynamic process, either transform into type II changes or remain type I. However, some paper found that type II changes can also transform into type I changes. Kuisma et al. [14] observed that 70 type II changes, and after 3 years, 10 (14.3%) became type I changes, and demonstrated that type II changes were not as stable as expected. Marshman et al. [19] also reported two cases of transformation from type II to type I. With the increase in age, the content of water and collagen in the nucleus pulposus is reduced, the ability to absorb shock and to buffer stress is weakened, and the degeneration of the intervertebral disk is gradually aggravated. Thus, age may be a factor for patients with type II changes.

### Gender and types

Our results show that gender is one of the factors leading to different types of Modic changes. Men patients are associated with type I changes, and women patients are associated with type II changes. Endplate microfractures may be a reason for it. Adams et al. [20] found that microfractures in the endplates and repair of trabecular bone are often seen in spinal cadaver specimens. The process of type I to type II transformation may be the repair process of endplate microfractures. Jensen et al. [21] conducted a prospective study of 100 patients with chronic LBP who had Modic changes, 50 of which underwent muscle function exercise and the other 50 underwent bed rest treatment. They found LBP relief in both groups, and there was no significant difference in the relief rates between groups. They thought that patients who underwent exercises also increase waist load, which is unbeneficial for the repair of microfracture. Although the muscle strength is enhanced, pain relief is unsatisfactory. It is generally believed that the workload of men patients is greater than women. Hence microfractures of men patients are less well repaired, which then results in persistent microfractures and inflammatory edema.

### Disk degeneration and types

Our results show that disk degeneration is one of the factors leading to the different types of Modic changes. We believe this may be related to the biomechanical effects of disk degeneration on the entire lumbar spine. Intervertebral disk is the largest bloodless tissue in the human body, it largely depends on the nutrients supplied by the cartilaginous endplate; the vascular channels in the cartilaginous endplate are particularly important for the nucleus pulposus [15]. Holm et al. [22] established a pig model of cartilage endplate injury by perforating the vertebral body, after 3 months, the content of water, aggrecan, type II collagen in annulus fibrosus was significantly lower compared to the normal group. Cinotti et al. [23] used the above-mentioned pig endplate injury model to draw a similar conclusion that endplate damage would lead to degeneration, and the degree of degeneration was positively correlated with the extent of endplate damage. Aggrecan and type II collagen are the main components of cartilage endplates, which can increase water content, absorb stress, and buffer shocks. They play an important role in the biomechanics of lumbar vertebrae, and their concentration is the main indicator for evaluating cartilage endplate degeneration. Modic changes are a manifestation of endplate biochemical changes on MRI and are early manifestations of endplate degeneration. The damages such as fissures and defects on the endplate also affect the nutrient supply of the intervertebral disk. When the intervertebral disk degenerates, the water content and type II collagen content in the nucleus pulposus tissue are reduced, and part of the nucleus pulposus can protrude to the posterior spinal canal, which weakens the stress absorption and shock buffering effect of the intervertebral disk against axial stress, resulting in a biomechanical change of the entire lumbar vertebrae. The vertebrae, therefore, withstand greater stress and eventually accelerate the damage to the endplate [12]. Severe degeneration of intervertebral disk means severe endplate damage, and hence type II changes often occur. Endplate degeneration and disk degeneration are closely related.

### Lumbar lordosis and types

Our results show that lumbar curvature is an important factor leading to the different types of Modic changes. Lumbar spine curvature is associated with type II changes, which we believe may be related to biomechanical factors. Lumbar lordosis plays a key role in maintaining the sagittal balance in the standing position of the body, and its curvature will have an effect on the biomechanics of the entire lumbar spine [24]. The presence of lumbar vertebral curvature disperses axial pressure, causing it to decompose into downward stress and forward shear. When the curvature of the lumbar spine is reduced, the ability to buffer axial stress is weakened, and the endplate and disk are subjected to more axial pressure. When the pressure is too large, the irreversible damage to the endplate can occur, resulting in the degeneration of the endplate and the intervertebral disk. Therefore, with a more severe degree of disk degeneration, type II changes occur more frequently, and with a milder degree of disk degeneration, type I changes occur more frequently. In the present study, we found that patients with type I changes mostly had disk degeneration of grades III and IV, while patients with type II changes mostly had disk degeneration of grades IV and V, which was consistent with changes in lumbar curvature. Tanaka et al. [25] studied the relationship between 140 vertebral bodies of 47 fresh corpses and lumbar stability and found that the stabilities of Pfirrmann grades III and IV vertebral segments were poor, while the stability of the vertebral segments of grade V was increased. Lao et al. [26] also used dynamic MRI to draw a similar conclusion, that the stability of the lumbar spine decreases with the degree of degeneration of the intervertebral disk, but when it reaches a certain level, it becomes stable again. Toyone et al. [7] studied 74 patients with Modic changes and found that 70% of patients with type I changes had significant lumbar instability, while only 16% of patients with type II changes had lumbar instability. Through the observation of this study, it was found that patients with type I changes had poorer lumbar stability than type II, and endplate microfracture was an important factor causing Modic type I changes, which may also be a cause of type II changes in patients with abnormal lumbar curvature.

### CAVE and types

Our results show that CAVE is one of the relevant factors leading to the different types of Modic changes. Small CAVE is related to type I changes, and large CAVE is related to the type II changes. We think this may be related to disk degeneration and difference in biomechanics. Endplate is a thin layer structure located between the vertebral body and disk, which can be divided into bony endplate and a cartilage endplate. The cartilage endplate is a part of the intervertebral disk, which is directly connected with the nucleus pulposus and annulus fibrosus, and serves to isolate the nucleus pulposus from contact with the vertebral cancellous bone. Wang et al. [27] used laser scanning technology to accurately measure the physiological morphological parameters of more than 500 lumbar vertebrae endplates. Endplate is the direct receiver site of disk transfer stress to the vertebral body, so the degree of CAVE plays an important role in the biomechanics of the lumbar spine. Under normal circumstances, the middle part of the endplate is the most stress-bearing area. When a disk degenerates, it will affect the stress distribution of the whole vertebral body, so that the periphery of the endplate will be under more pressure, resulting in the change of endplate morphology [28]. When the axial pressure of the bony endplate is increased, the body can increase the sagittal diameter of the vertebral body by flattening the endplate, thereby reducing the pressure per unit area. This self-adaptive change can be considered as a self-protection mechanism of the endplate [28]. When endplate degenerates, the content of aggrecan and type II collagen decreases, and endplate loses its elasticity and self-repairing ability. Endplate loses its original concave structure under chronic axial pressure load and flattens. Therefore, the shape of endplate can not only reflect the degeneration of the endplate itself but also reflect the degree of degeneration of the disk.

## Conclusions

In conclusion, type II changes are most common in patients with LBP, followed by type I, and type III is the least common. Modic changes occur mostly in L4/5 and L5/S1 segments. Age, gender, disk degeneration, lumbar curvature, VACE was associated with different types, among these factors, gender, and lumbar curvature have the strongest correlations. Therefore, for patients with type I changes, we believe that simple conservative treatment can achieve satisfactory results, and an extended period of conservative treatments is recommended. For patients with type II changes, when conservative treatments cannot achieve satisfactory effects, other aggressive treatment options can be taken to prevent further disease progression. The study is a preliminary analysis, and the specific clinical efficacy needs further investigation.

## Data Availability

The data used to support the findings of this study were supplied by Jun Zou under license and so cannot be made freely available. Requests for access to these data should be made to Jun Zou, Department of Orthopaedic Surgery, The First Affiliated Hospital of Soochow University 188 Shizi St. Suzhou, China, 215006, Email: jzou@suda.edu.cn
